# Effects of *lactobacillus plantarum* ZJ316 on pig growth and pork quality

**DOI:** 10.1186/1746-6148-8-89

**Published:** 2012-06-25

**Authors:** Xiaona Wang, Xiuyu Lou, Dafeng Song, Xin Wang, Qing Gu

**Affiliations:** 1 Department of Biotechnology, College of Food Science and Biotechnology, Zhejiang Gongshang University, No. 149, Jiaogong Road, Hangzhou, 310035, China; 2 Institute of Plant Protection and Microbiology, Zhejiang Academy of Agricultural Sciences, No. 198, Shiqiao Road, Hangzhou, 310021, China

**Keywords:** Probiotics, *Lactobacillus plantarum*, Pig, Pork quality

## Abstract

**Background:**

*Lactobacillus plantarum* is a plant-associated bacterial species but it has also been found in human, mouse and porcine gastrointestinal tracts. It can ferment a broad spectrum of plant carbohydrates; it is tolerant of bile salts and low pH, and it has antagonistic potential against intestinal pathogens. However, experiments reporting the use of *L. plantarum* as a probiotic are limited. In this study, the effects of *L. plantarum* ZJ316 isolated from infant fecal samples on pig growth and pork quality were investigated.

**Results:**

One hundred and fifty newly weaned pigs were selected randomly and divided into five groups. Group 1 was fed a diet supplemented with the antibiotic mequindox; Groups 2, 3 and 4 were fed a diet supplemented with *L. plantarum* and no antibiotic; and Group 5 was fed a mixture of mequindox and *L. plantarum.* After a 60 days initial treatment, samples were collected for evaluation. The results showed that, the *L. plantarum* ZJ316 has probiotic effects on pig growth and that these effects are dose dependent. The effects of a dose of 1 × 10^9^ CFU/d were more pronounced than those of a dose of 5 × 10^9^ CFU/d or 1 × 10^10^ CFU/d. In Group 2 (1 × 10^9^ CFU/d), the diarrhea (p = 0.000) and mortality rates (p = 0.448) were lower than in antibiotic-treated pigs (Group 1), and the daily weight gain (p = 0.001) and food conversion ratios were better (p = 0.005). Improved pork quality was associated with *Lactobacillus* treatment. pH (45 min, p = 0.020), hardness (p = 0.000), stickiness (p = 0.044), chewiness (p = 0.000), gumminess (p = 0.000) and restoring force (p = 0.004) were all significantly improved in *Lactobacillus*-treated pigs (Group 2). Although we found that *L. plantarum* exerted probiotic effects on pig growth and pork quality, the mechanisms underlying its action require further study. Polymerase chain reaction-denaturing gradient gel electrophoresis results showed that the gut bacterial communities in *Lactobacillus*- and antibiotic-treated pigs were very similar and the quantity of *L. plantarum* ZJ316 was below the detection limits of DGGE-band sequencing. The concentration of short-chain fatty acids in *Lactobacillus*- and antibiotic-treated fecal samples were not significantly different (p = 0.086). However, the villus height of ilea (p = 0.003), jejuna (p = 0.000) and duodena (p = 0.036) were found to be significantly improved by *Lactobacillus* treatment.

**Conclusion:**

*L. plantarum* ZJ316 was found to have probiotic effects, improving pig growth and pork quality. The probiotic mechanism might not involve *L. plantarum* colonization and alteration of the gut bacterial community. Rather, it might be related to the inhibition of the growth of opportunistic pathogens and promotion of increased villus height.

## Background

Weaning stress can destroy the balance of intestinal microbiota in young mammals. Such periods of stress may allow opportunistic pathogens to multiply and cause gastrointestinal (GI) disorders [1]. To promote growth and reduce the incidence of diarrhea, sub-therapeutic antibiotics have been widely used in pig diets [2]. However, this procedure has public health consequences because of the high risk of the development of antimicrobial resistance among pathogenic bacteria, which may be then transferred to humans [3,4]. It is therefore necessary to find ways to replace antibiotics in pig feeding strategies.

Several members of *Lactobacillus*, which is part of the normal mucosal microbiota of pigs, have been found to be good probiotics [5,6]. *Lactobacillus* and similar bacteria, as well as their metabolites, can control pathogens, such as *Escherichia coli*[7,8], *Salmonella typhimurium*[9], and others. Selected strains of *L. plantarum* possess properties that make them promising candidates for probiotics in feed additives [10]. However, experiments reporting the use of *L. plantarum* as a probiotic are limited. Pieper et al. determined whether a single administration of *L. plantarum* DSMZ 8862/8866 either before or at the time of weaning can affect the intestinal microbiota of pigs. The results showed that *L. plantarum* DSMZ 8862/8866 has positive results on GI health [11]. The effects were found to be better when the probiotics were administered at the time of weaning than when they were administered before weaning.

Due to the high mortality of pigs after removing antibiotics at weaning (unpublished data), most farmers are reluctant to accept this option [12]. In this study, we determined whether *L. plantarum* could replace commonly used antibiotics. We also investigated the effects of *L. plantarum* ZJ316 on pig growth, pork quality, gut morphology and gut microbiota.

## Results

### Inhibitory effects of *L. Plantarum* ZJ316 culture supernatants

As shown in Table 1, the culture supernatants of *L. plantarum* ZJ316 had strong inhibitory effects on some pathogenic bacteria, such as *Salmonella*, *Escherichia coli*, and *Listeria monocytogenes*. However, probiotics such as *L. fermentum*, *Lactococcus lactis* and *Saccharomyces cerevisiae* were not inhibited by the culture supernatants of *L. plantarum* ZJ316. These results showed that the strain *L. plantarum* ZJ316 may be a good probiotic. As shown in Additional file 1 Figure S1, the supernatants still exhibited an inhibitory effect on *Escherichia coli* and *Salmonella* after the pH was adjusted to 6.0. This observation indicates that the inhibitory effect was not only due to the low pH. We also investigated the effects of two main short-chain fatty acids on bacterial inhibition. The results showed that at pH 3.5, acetic acid and lactic acid cannot inhibit the growth of indicator bacteria (Additional file 1 Figure S2).

**Table 1:** **Inhibition spectrum of culture supernatants of *L. plantarum *ZJ316**

**Bacterial name**	Inhibitory effects^*^
*Acetic bacteria*	-
*Bacillus subtilis*	+
*E. coli*	+++
*Lactobacillus fermenti*	-
*Lactobacillus plantarum ZJ316*	-
*Lactococcus lactis*	-
*Listeria monocytogenes*	++
*Micrococcus luteus*	+
*Pseudomonas aeruginosa*	+
*Pseudomonas putida*	++++
*Shigella flexneri*	++
*Saccharomyces cerevisiae*	-
*Salmonella*	++
*Shigella dysenteriae*	+
*Staphylococcus aureus*	+
*Staphylococcus citreus*	+
*Vibrio parahaemolyticus*	-

*, Inhibition zone in diameter (mm): +++, 19-24; ++, 15-18; +, 10-15; -, no observed inhibit effect.

### Effects of *L. Plantarum* ZJ316 on pig growth

As shown in Table 2, different treatments affected pig growth. Compared to group 1 (which was treated with mequindox), other groups treated with *Lactobacillus* or treated with both showed better pig growth. The average daily gain and food conversion ratio was higher improved in the *Lactobacillus*-treated groups than in Group 1. Group 2 showed significantly better growth performance than Group 1. Diarrhea ratios were significantly lower in *Lactobacillus*-treated groups than in Group 1. The effect of *Lactobacillus* on pig growth varied according to dosage. The effects in Group 2 (1 × 10^9^ CFU/day) were more pronounced than in Group 3 (5 × 10^9^ CFU/day) or Group 4 (1 × 10^10^ CFU/day). In general, mequindox was found to improve the probiotic effects of high dosages of *Lactobacillus*. In Groups 4 and 5, pigs were given the same dose of *Lactobacillus* (1 × 10^10^ CFU/day) orally. Mequindox was added to the diet fed to pigs in Group 5. The results showed that mequindox affected the probiotic effects on pig growth, but these effects were not significant.

**Table 2:** **Effects of different treatments on pig growth parameters, mortality and prevalence of diarrhea**^**#**^

	**Group 1**	**Group 2**	**Group 3**	**Group 4**	**Group 5**
Food intake (g/day)	974.21 ± 5.51^a^	911.42 ± 34.50^ab^	864.81 ± 43.78^b^	901.39 ± 44.51^ab^	860.80 ± 19.73^b^
Daily gain (g/day)	454.90 ± 18.14^a^	520.30 ± 9.29^b^	470.20 ± 13.27^a^	477.50 ± 13.87^a^	469.30 ± 10.31^a^
Food conversion ratio	2.23 ± 0.08^a^	1.76 ± 0.12^b^	1.85 ± 0.15^b^	1.89 ± 0.04^b^	1.83 ± 0.02^b^
Mortality (%)	10.00 ± 10.00 ^a^	3.33 ± 3.33^a^	3.33 ± 3.33 ^a^	6.67 ± 6.67 ^a^	3.33 ± 3.33^a^
Diarrhea (%)	7.06 ± 0.66 ^a^	2.17 ± 0.10 ^b^	2.28 ± 0.20^b^	3.39 ± 0.78 ^bc^	4.28 ± 0.22^c^

^#^Group 1 represents pigs treated with antibiotics 105 mg/day. Groups 2, 3, and 4 represent pigs treated with *L. plantarum* ZJ316 1 × 10^9^ CFU/d, 5 × 10^9^ CFU/d, and 1 × 10^10^ CFU/d, respectively. Group 5 represents pigs treated with antibiotics 105 mg/d and *L. plantarum* ZJ316 1 × 10^10^ CFU/d . All values are expressed as mean ± standard error.

^a, b and c^ Different superscript letters within a row represent statistically significant differences among groups (p < 0.05).

The effects of *L. plantarum ZJ316* on growth was more pronounced in Group 2 than in Groups 3, 4, or 5. Only pigs in groups 1 and 2 were selected for further analysis.

### Effects of *L. Plantarum* ZJ316 on pork quality

Three pigs selected randomly from Group 1, and six pigs selected randomly from Group 2 were killed for evaluation of pork quality. In Group 2, three pigs were killed after cessation of addition of *Lactobacillus* to their diet. These pigs are here referred to as Group 2-1. The remaining three pigs in Group 2 were killed one week after cessation of the addition of *Lactobacillus*, and are here referred to as Group 2-2. As shown in Table 3, the pork quality was different between antibiotic- and *Lactobacillus*-treated pigs. The pH (45 min) and several meat texture evaluation parameters, such as hardness, stickiness, chewiness, gumminess and restoring force were significantly improved by *Lactobacillus* (Group 2-1) compared with antibiotic-treated pigs. More interesting is the fact that the pork quality had some changes one week after stopping *Lactobacillus* treatment. The lightness, ∇E, drop loss (24 h), hardness, stickiness, chewiness, gumminess and restoring force of *longissimus* were significantly changed one week after cessation of the inclusion of *Lactobacillus*.

**Table 3:** **Parameters used for evaluation of pork quality**^**#**^

		**Group 1**	**Group 2-1**	**Group 2-2**
Longissimus muscle color	Lightness	39.06 ± 1.13^ab^	41.58 ± 1.71^a^	35.39 ± 0.96^b^
	Redness	5.10 ± 0.92^a^	5.95 ± 0.11^a^	4.48 ± 0.70^a^
	Yellowness	1.88 ± 0.29^a^	2.42 ± 0.30^a^	1.57 ± 0.41^a^
	∇E	55.72 ± 1.04^ab^	52.47 ± 2.51^a^	59.97 ± 0.93^b^
Fillet color	Lightness	49.60 ± 5.03^a^	45.82 ± 3.39^ab^	39.08 ± 0.40^b^
	Redness	10.93 ± 0.92^a^	11.16 ± 0.85^a^	9.11 ± 0.61^a^
	Yellowness	3.73 ± 0.99^a^	3.18 ± 0.57^a^	2.07 ± 0.06^a^
	∇E	47.04 ± 4.64^a^	50.66 ± 3.14^ab^	56.86 ± 0.36^b^
pH value	45 minutes	6.25 ± 0.01^a^	6.38 ± 0.03^b^	6.39 ± 0.04^b^
	24 hours	5.82 ± 0.04^a^	5.84 ± 0.02^a^	5.83 ± 0.03^a^
Drip loss (%)	24 hours	5.95 ± 0.94^a^	5.70 ± 1.01^a^	2.84 ±0.30^b^
	48 hours	3.28 ± 0.42^a^	2.80 ± 0.69 ^ab^	1.70 ± 0.29^b^
Marbling score		3.02 ± 0.12^a^	3.25 ± 0.12^a^	3.25 ± 0.12^a^
Hardness (g)		209.43 ± 16.50^a^	138.47 ± 6.70^b^	75.03 ± 7.27^c^
Stickiness (g·s)		-4.43 ± 0.39^a^	-6.88 ± 1.30^b^	-2.82 ± 0.38^a^
Springiness		1.01 ± 0.02^a^	0.96 ± 0.03^a^	1.09 ± 0.08^a^
Chewiness		152.14 ± 13.50^a^	93.51 ± 8.57^b^	61.35 ± 5.45^c^
Gumminess		153.96 ± 10.27^a^	97.80 ± 10.35^b^	55.59 ± 7.61^c^
Cohesiveness		0.73 ± 0.01^a^	0.71 ± 0.02^a^	0.73 ± 0.01^a^
Restoring force		0.46 ± 0.03^a^	0.35 ± 0.02^b^	0.42 ± 0.02^a^

^#^Group 1 represents samples collected from pigs treated with antibiotics 105 mg/d. Group 2 represents samples collected from pigs treated with *L. plantarum* ZJ316 1 × 10^9^ CFU/d. Group 2-1 represents samples collected immediately after the cessation of *L. plantarum* and Group 2-2 represents samples collected one week after the cessation of *L. plantarum.* All values represent mean ± standard error.

^a, b and c^ Different superscript letters within a row represent statistically significant differences among groups (p < 0.05).

### Histologic alterations of intestinal ileal mucosa

No features suggesting histopathology in the ilea, jejuna and duodena of antibiotic- and *Lactobacillus*-treated pigs were observed at any time during this study. The intestinal mucosa was regularly organized in intestinal villi and crypts in both groups. The height and thickness of the villi differed across treatment groups. In the *Lactobacillus*-treated group, the height of the villi was greater and the density was thicker than in the antibiotic-treated group. After microscopic observation, villus height and crypt depth were quantified using a Leica MZ16A (Leica, Germany). The ratio of villus height to crypt depth was calculated and the results are listed in Table 4. The results showed that the villus height in the ilea, jejuna and duodena of *Lactobacillus*-treated pigs (Group 2-1) was significantly greater than that of antibiotic-treated pigs (Group 1). In group 2-2, the villus height in the duodena and the crypt depth in the jejuna were significantly greater one week after cessation of *Lactobacillus* exposure.

**Table 4:** **The effects of *Lactobacillus *on gut villus height (μm) and crypt depth (μm)**^**#**^

		**Group1**	**Group2-1**	**Group2-2**
Ileum	Villus height (VH)	479.81 ± 19.07^a^	557.92 ± 19.99^b^	532.83 ± 15.34^b^
	Crypt depth (CD)	263.91 ± 12.56^a^	226.01 ± 13.24^b^	202.92 ± 7.46^b^
	VH/CD^*^	1.89 ± 0.09^a^	2.71 ± 0.17^b^	2.74 ± 0.13^b^
Jejunum	Villus height (VH)	411.18 ± 14.67^a^	504.54 ± 14.06^b^	534.72 ± 13.49^b^
	Crypt depth (CD)	280.13 ± 14.89^a^	264.14 ± 15.83^a^	222.80 ± 10.54^b^
	VH/CD	1.64 ± 0.14^a^	2.07 ± 0.11^b^	2.61 ± 0.17^c^
Duodenum	Villus height (VH)	410.10 ± 11.24^a^	453.35 ± 13.17^b^	576.80 ± 17.85^c^
	Crypt depth (CD)	221.23 ± 10.11^a^	212.45 ± 10.79^a^	217.98 ± 10.60^a^
	VH/CD	2.00 ± 0.13^a^	2.28 ± 0.12^a^	2.88 ± 0.19^b^

^#^Group 1 represents samples collected from pigs treated with antibiotics 105 mg/d. Group 2 represents samples collected from pigs treated with *L. plantarum* ZJ316 1 × 10^9^ CFU/d. Group 2-1 represents samples collected immediately after the cessation of *L. plantarum* and Group 2-2 represents samples collected one week after the cessation of *L. plantarum.* All values represent mean ± standard error.

^*^ Represents the ratio of villus height to crypt depth.

^a, b and c^ Different superscript letters within a row represent statistically significant differences among groups (p < 0.05).

### Changes in the gut bacterial community due to different treatments

**Figure 1:**
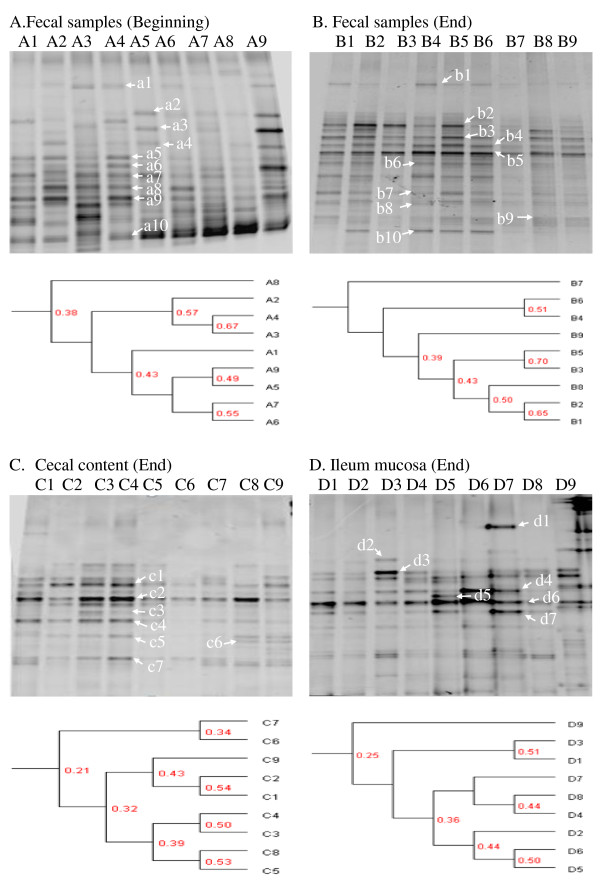
**Polymerase chain reaction-denaturing gradient gel electrophoresis (PCR-DGGE) analysis of the gut bacterial community after different treatments.** PCR-DGGE of the 16 S rDNA V3 region was performed to analyze the bacterial community. Similarities were assessed by cluster analysis using Quantity One. (**A**) Nine fecal samples were randomly collected at the beginning of the experiment. (**B**, **C**, and **D**) Fecal, cecal, and ileal samples collected at the end of the experiment. B1–B3, B4–B6, and B7–B9 represent fecal samples collected from groups 1, 2-1, and 2-2, respectively. C1–C3, C4–C6, and C7–C9 represent cecal contents collected from groups 1, 2-1, and 2-2, respectively. D1–D3, D4–D6, and D7–D9 represent ileal mucosa collected from groups 1, 2-1, and 2-2, respectively. In Group 1, mequindox was added to the subjects’ diet. In Group 2-1, *L. plantarum* ZJ316 was added and mequindox was removed, and samples were collected immediately after halting the addition of *L. plantarum ZJ316* (day 95). In Group 2-2, *L. plantarum* ZJ316 was added and mequindox was removed, and samples were collected one week after the cessation of addition of *L. plantarum* ZJ316 (day 102).

DGGE profiles of PCR products of the 16 S rRNA gene V3 region from pig fecal, ileal mucosa and cecal contents revealed that the overall bacterial community was not significantly changed by *Lactobacillus* treatment (Group 2-1 and 2-2) compared to antibiotic-treated (Group 1). Before starting this experiment, the bacterial community of pig fecal samples were randomly distributed and varied due to individual animal differences (Figure 1A). The patterns of DGGE bands in fecal, cecal and ileal samples were similar between antibiotic- and *Lactobacillus*-treated groups (Figures 1B, 1C and 1D). To obtain an objective interpretation of the electrophoretic patterns of the different treatment groups, the samples were subjected to a numerical analysis based on the Dice similarity coefficient, then used for cluster analysis. As shown in Figure 1, the cluster analysis results indicated that the bacterial community in fecal, cecal and ileal samples did not show major changes between antibiotic and *Lactobacillus* treatment. Samples taken from different treatment groups were distributed randomly in the cluster tree.

To determine whether the *L. plantarum* ZJ316 colonized in pigs’ guts, most of the single DGGE bands were excised for cloning and sequencing. Three clones were selected randomly from each band for sequencing. The sequences were then identified using a Blast search. The bacteria with highest value for each band are listed in Table 5. No bands showing significant similarity to *Lactobacillus* appeared before the start of treatment. At the end of this study, we observed two bands from fecal samples, two bands from cecal contents and 1 band from ileum mucosa that showed pronounced similarity to *Lactobacillus*. No band showed pronounced similarity to *L. plantarum.*

**Table 5:** Identification of PCR-DGGE bands using cloning and sequencing

**No.**	**Similar species**	**No.**	**Similar species**
a1	*Erysipelothrix rhusiopathiae*	b1	*Eothenomys smithi* HEG193, *Streptococcus hyointestinalis*
a2	*Aerococcus urinaeequi*, *Weissella paramesenteroides*	b2	*Enterobacteriaceae bacterium*, *Streptococcus hyointestinalis*, *Blautia glucerasea*
a3	*Anaerococcus tetradius*, *Clostridium* sp.	b3	*Rumen bacterium* NK4B114, *Clostridium cadaveris*, *Lactobacillus gallinarum*
a4	*Ruminococcus obeum*	b4	*Lachnospira multipara*, Bacterium YE62, *Acetivibrio ethanolgignens*
a5	*Eubacterium eligens*, *Clostridiales bacterium*	b5	*Streptococcus gallolyticus* subsp.
a6	*Rumen bacterium* NK4B114	b6	*Faecalibacterium prausnitzii*
a7	*Lachnospiraceae bacterium*, *Blautia glucerasea*	b7	*Blautia glucerasea*
a8	*Anaerostipes butyraticus*, *Gemmiger formicilis*, *Ruminococcus obeum*	b8	*Peptoniphilus* sp., *Faecalibacterium prausnitzii*
a9	*Eubacterium hadrum*, Bacterium YE257	b9	*Peptococcus* sp., *Anaerococcus tetradius*
a10	*Clostridium cadaveris*	b10	*Lactobacillus johnsonii*
c1	*Lactobacillus acidophilus*, *Ruminococcus* sp., *Lactobacillus amylolyticus*	d1	*Clostridium sordellii*, *Lachnospira multipara*
c2	*Rumen bacterium* NK4B114, *Streptococcus gallolyticus* subsp.	d2	*Lysinibacillus* sp.
c3	*Ruminococcus obeum*	d3	*Clostridium nexile*, *Klebsiella* sp., *Clostridiales bacterium*
c4	*Gemmiger formicilis*	d4	*Eothenomys smithii*, *Lactobacillus gasseri*, *Clostridium cadaveris*
c5	*Actinomycetales bacterium*, *Ruminococcus obeum*, *Lactobacillus vaginalis*	d5	*Enterococcus faecium*
c6	Blautia glucerasea, Bacterium YE257	d6	*Streptococcus gallolyticus* subsp.
c7	*Clostridiales bacteriumClostridium sordellii*	d7	*Peptostreptococcus* sp., *Staphylococcus* sp., *Eubacterium* sp.

### Changes in the concentration of short-chained fatty acids (SCFAs) in pig fecal samples

Although the concentration of most SCFAs was greater in the *Lactobacillus*-treated group (Group 2), the difference between antibiotic- and *Lactobacillus*-treated groups was not significant (Table 6).

**Table 6:** **Concentration of short-chain fatty acids in pig fecal samples**^**#**^

	**Group 1**^&^	**Group 2**^&^	**p-value**^§^
Formic acid	8.45 ± 1.13	11.68 ± 0.39	0.053
Tartaric acid	0.48 ± 0.04	0.44 ± 0.05	0.563
Malic acid	0.35 ± 0.07	0.48 ± 0.09	0.339
Lactic acid	0.52 ± 0.07	0.53 ± 0.01	0.921
Acetic acid	7.66 ± 1.80	10.87 ± 2.71	0.379
Citric acid	1.76 ± 0.92	4.33 ± 2.33	0.363
Propionic acid	2.47 ± 0.47	3.04 ± 0.15	0.317
Butyric acid	24.92 ± 1.01	28.57 ± 4.96	0.511
Isovaleric acid	1.88 ± 0.60	3.49 ± 2.18	0.514
Total	48.49 ± 5.38	63.43 ± 3.79	0.086

^#^Group 1 represents samples collected from pigs treated with antibiotic 105 mg/d; Group 2 represents samples collected from pigs treated with *L. plantarum* ZJ316 1 × 10^9^ CFU/d. And all values represent mean ± standard error

&, represents the concentration of short-chain fatty acids (mMol) in each gram of fecal sample.

§, represents the p-value of statistically significant difference between groups 1 and 2.

### The inhibitory effects of mequindox on *L. Plantarum* ZJ316

**Figure 2:**
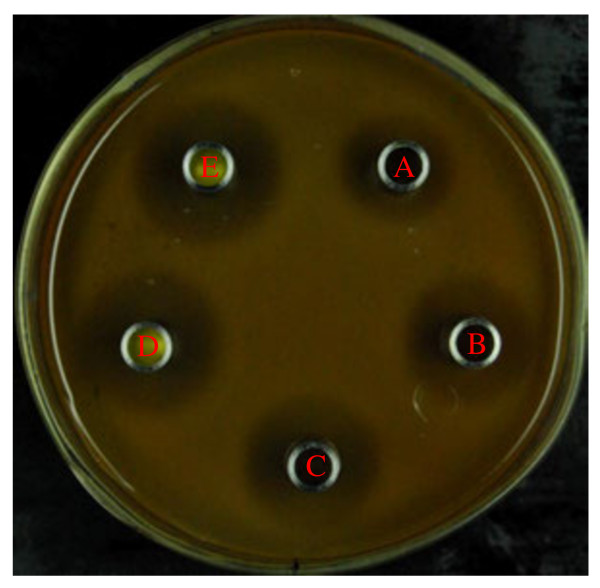
**Inhibitory effects of mequindox on *L. plantarum *ZJ316.** An Oxford cup test was executed. The inhibitory effects of (**A**) 50 μg/ml, (**B**) 100 μg/ml, (**C**) 250 μg/ml, (**D**) 500 μg/ml and (**E**) 750 μg/ml of mequindox were evaluated.

As shown in Figure 2, the growth of *L. plantarum* ZJ316 was affected by adding 50 μg/ml, 100 μg/ml, 250 μg/ml, 500 μg/ml and 750 μg/ml of mequindox, and the inhibitory effects were found to be dose-dependent. The high concentration of mequindox had strong inhibitory effect on the growth of *L. plantarum* ZJ316.

## Discussion

Results of this study showed that *L. plantarum* ZJ316, isolated from infant fecal samples, had a good inhibitory effect on some pathogenic bacteria *in vitro*. And *L. plantarum* ZJ316 can significantly improve pig growth at a dose of 1 × 10^9^ CFU/d (Group 2). The probiotic effects of *L. plantarum* ZJ316 are consistent with those observed in previous studies. Foo et al. reported that the effects of feeding *Lactobacillus* species I-UL4 and their metabolites to weaned rats can improve growth [13,14]. Thanh et al. showed that metabolites produced by *L. plantarum* RS5, RI11, RG14 and RG11 strains can improve chicken growth [15].

Although the effects of probiotics usage in pigs are not always consistent, beneficial effects have been documented [16-18]. The mode of action of a given probiotic may include modulation of host microflora [19,20], modifications of the morphology of the intestinal epithelium [21], regulation of the host immunity system [22], and the concentration of gut SCFAs, such as acetate [23]. In this study, we investigated the change in gut bacterial community, morphology of ileal mucosa, and the concentration of gut SCFAs due to treatment with *L. plantarum* ZJ316.

As shown in Figure 1, the bacterial communities in antibiotic- and *Lactobacillus*-treated groups (Groups 1, 2-1 and 2-2) were very similar. These results showed that the community of gut bacteria was not significantly altered by treatment with *L. plantarum* ZJ316. Some previous researches have shown similar results. Ohashi et al. showed only a slight change in bacterial communities attributable to orally administered *Lactobacillus casei* strain Shirota (LCS) [24]. Su et al. also reported no remarkable changes in the overall microbial community in the hind gut after orally administrated *L. sobrius* S1 [25]. In this study, there are three possible explanations for explain this phenomenon. The first is that the pigs may have developed a stable microbiota after weaning, and this microbiota may be hard to change. Although the porcine GI tract harbors a highly diverse microbial ecosystem [26], Konstantinov et al. reported that once the gut microbiota has matured, it can remain stable for a long time [27]. Second, probiotics, known widely as beneficial bacteria and yeasts, assist in the restoration of normal levels of beneficial microorganisms without destroying the bacterial communities of the GI tract. Third, the ability of this *Lactobacillus* to colonize the gut epithelium may be low, because the bacterial species used in this experiment was isolated from infant fecal samples. DGGE sequencing results verified this. 34 prominent DGGE bands were extracted for sequencing and the results are listed in Table 5. We can see that, although five bands maybe related to *Lactobacillus* species, no *L. plantarum* was found in any of them. Dunne et al. reported that permanent persistence of an allochthonous strain in the host GI microbiota was virtually impossible [28]. *L. plantarum* is not the predominant *Lactobacillus* in pigs. *L. sobrius* was found to be the most dominant species of *Lactobacillus* in pigs in both pre- and post-weaning [27,29,30]. Previous results have also demonstrated that ingested probiotic strains do not become established members of the normal microbiota but persist only for a short time [31-34]. There is also evidence that common probiotic strains differ in their degree of persistence [31,35].

Villi are important components of the digestive tract. They are involved in the absorption of nutrients from the small intestine. The condition of intestinal villi and epithelial cells on the apical surface of the villi is known to be a reliable indicator of the enteral nutrient absorption of feed ingredients in chickens [36] and pigs [37]. However, during post weaning, pigs commonly suffered morphologic atrophy and crypt hyperplasia, which can limit the absorption of voluntary feed intake and weight gain after weaning [38,39]. One characteristic of an effective probiotic is to increase the villus height. SCFAs were considered to be the main factors for stimulating the development of intestinal mucosa [40]. Although the concentration of SCFAs was not significantly different between antibiotic- and *Lactobacillus*-treated groups (Group 1 and Group 2) in this study (Table 6), the villus height and crypt depth both improved after treatment with *L. plantarum* ZJ316*.* There may be other mechanisms by which probiotics can improve intestinal epithelial and villi. It has been established that the effects of probiotic bacteria may also result from soluble factors that can alter epithelial permeability [41]. Two soluble proteins, p40 and p75, were purified from the culture supernatant of *Lactobacillus rhamnosus* GG (LGG), can prevented TNF-induced apoptosis and intestinal barrier disruption in colonic epithelial cells [42]. The same group also reported that the probiotic LGG can prevent cytokine-induced apoptosis in colon cells through activation the pathways of anti-apoptotic Akt and protein kinase B, and inactivation of the pathway of pro-aptoptic p38 mitogen-activated protein kinase [43].

The effect of *L. plantarum* ZJ316 on pig growth was found to be dose-dependent. The effects of a dose of 1 × 10^9^ CFU/d were more pronounced than those of doses of 5 × 10^9^ CFU/d and 1 × 10^10^ CFU/d. This may be related to cross-talk between the probiotics and the host’s immune system. Besides these anti-infective properties, probiotics also act upon the immune and inflammatory response. Probiotic supplementation can enhance SIgA production in both rodents and humans [44]. This appears to be a paradox. Probiotics and nonpathogenic commensals can boost the overall SIgA antibody response, while SIgA can trigger intestinal exclusion and subsequent elimination [45-48]. A high dose of probiotics may induce a strong immune and stress response. Rodrigues et al. reported that germ-free mice colonized with *S. boulardii* displayed more pronounced anti-*S. boulardii* IgA expression than un-colonized mice [49]. This dose-dependent effect of probiotics has also been observed by other researchers. A change in levels of low-density lipoprotein (LDL) and high-density lipoprotein (HDL) in the blood due can be attributed to the administration of *Bifidobacterium animalis* subsp lactis (BB-12) and *Lactobacillus paracasei* subsp paracasei (CRL-431) was dependent on a specific dose of 10^8^ for LDL and 10^9^ for HDL [50]. A daily dose of *Lactobacillus rhamnosus* GR-1 plus *Lactobacillus fermentum* RC-14 at 1.6 × 10^9^ has a better success of restoring and maintaining a normal vaginal flora than doses of 8 × 10^8^ and 6 × 10^9^[51]. There may be some other mechanisms that may explain this phenomenon. In another study, they found that low bacteria/DC (dendritic cells) ratio better regulates the effects on DCs *in vitro*, suggesting that different intracellular signaling pathways become activated when bacteria are present at high doses [52]. Considering the dose-dependent nature of the effects of *L. plantarum* ZJ316, this may be the reason why mixed treatment group (Group 5, 105 g mequindox and 1 × 10^10^ *L. plantarum* ZJ316 CFU/d) experienced a better probiotic effect than the same dose of antibiotic and probiotic administered alone (Group 1 and Group 4). As shown in Table 2, although the differences between Groups 4 and 5 were not significant, the parameters used for evaluating growth performance had better values for Group 5 than for Groups 1 and 4. These values were similar to those of the lower dose group (Group 3, 5 × 10^9^ *L. plantarum* ZJ316 CFU/d). As shown in Figure 2, mequindox inhibited the growth of *L. plantarum* ZJ316. This shows that mixtures of mequindox and *L. plantarum* ZJ316 may affect the effective dose of *L. plantarum* ZJ316. This may be why the effects of mixture group more similar to lower dose group (Group 3). Other researches also showed that antibiotics can affect the effects of bacteria-derived probiotics, and should be separated from antibiotics by at least two hours [53].

Here, treatment with *L. plantarum* also improved pork quality. Significant improvements were observed in Group 2 with regards to hardness, stickiness, chewiness, gumminess, and restoring force. This may meet the nutritional needs of human consumers. Until now, reports on the effects of probioitcs on pork quality have been rare; however, further study is needed.

## Conclusion

The mechanism whereby *L. plantarum* ZJ316 improved pig growth and pork quality may not be through *L. plantarum* colonization and alteration of the gut bacterial community. The promotion of pig growth and pork quality may rather be related to the metabolites inhibiting the growth of opportunistic pathogens and increasing the villus height.

## Methods

### Animals and experimental design

One hundred and fifty Landrace-Yorkshire pigs were randomly selected from 26 sows. The ratio of young boars to gilts was about 1:1 and the young boars were castrated at 2 weeks. Pigs received no creep feed and were weaned at an age of 28 days. After weaning, pigs were randomly divided into five groups and each group was kept in three pens. In each pen, 10 pigs were selected from 4–5 sows and were kept under standard conditions (natural light regime, humidity of 50–60%, at a temperature of 25 ± 1 °C) at the experimental pig farm of Zhejiang Academy of Agricultural Sciences. Before this study, pigs were raised one week with antibiotic (mequindox) to adjust to the new environment. At weaning, the average pig weight was 7.69 ± 0.82 kg, and at the beginning of this experiment on day 35, the average was 11.59 ± 1.42 kg. During this experiment, all pigs were fed the same basic diet (Table 7) *ad libitum*. Swine fever vaccine, foot and mouth vaccine, pseudorabies vaccine, and porcine reproductive and respiratory syndrome vaccine were used during this period. In order to determine whether the strain of *L. plantarum* ZJ316 had any ability to replace the antibiotics, Group 1 was fed a diet containing mequindox; Groups 2, 3 and 4 were fed a diet without antibiotics but with *L. plantarum* ZJ316; and Group 5 was fed a mixture of mequindox and *L. plantarum* ZJ316*.* The antibiotic was mixed with feeds and the *L. plantarum* ZJ316 was administered in drinking water. The concentration of antibiotic used for each pig in groups 1 and 5 was 105 mg/d, and the average concentrations of *L. plantarum* ZJ316 for each pig in Group 2, 3, 4 and 5 were 1 × 10^9^ CFU/d, 5 × 10^9^ CFU/d, 1 × 10^10^ CFU/d and 1 × 10^10^ CFU/d, respectively. In order to control the concentrations of antibiotics and *L. plantarum*, small portions of food and water containing mequindox or *L. plantarum* were fed to pigs before free feeding. The antibiotic and *L. plantarum* ZJ316 were given to these pigs every day until the end of this experiment (i.e.*,* 60 d later). We did not use un-treated pigs as controls because the morbidity and mortality rates are very high among these pigs (more than 20% in before experiments) and the pathogenicity may easily be transferred to other pigs, causing great economic losses.

**Table 7:** Composition of the diet used in this study

**Ingredients**	**g·kg-1**	**Nutritional level**	
Corn	645	Crude protein, %	18.23
Soy bean meal	250	Digestible energy (Mcal/kg)	3.15
Wheat middlings	50	Lysine (%)	1.13
Fish meal	20	Methionine (%)	0.36
Limestone	10.6	Threonine (%)	0.78
Additive^a^	10	Calcium (%)	0.76
Calcium Bicarbonate	5	Phosphorous (%)	0.61
Salt	3.5		
L-lysine	2		
Choline chloride	1		
Fromadriamycin	0.8		
LysoforteTM Dry	0.7		
Threonine	0.5		
Methionine	0.5		
Capsozyme	0.3		
Phytate	0.1		

^a^Additive (amount/kilogram of diet): Ca, 849 mg; Zn, 150 mg; Fe, 132 mg; Mn, 20 mg; Cu, 12 mg; Se, 0.31 mg; I, 0.79 mg; vitamin A, 1,298 IU; vitamin D3, 260 IU; vitamin E, 2.4 IU; menadione (sodium bisulphate), 0.143 mg; vitamin B12, 3.3 mg; riboflavin, 0.880 mg; d-pantothenic acid, 2.6 mg; niacin, 4.4 mg.

All pigs used in these experiments were treated according to the current regulation of laboratory animal management in China and approved by the laboratory animal care and usage committee, Zhejiang Academy of Agricultural Sciences.

### Isolation and identification of *L. Plantarum* ZJ316

*Lactobacillus* was isolated from an infant fecal samples using Man–Rogosa–Sharpe (MRS) medium (Hopebio, Qingdao, China). In brief, fecal suspension was spread on MRS agar plates and cultured overnight at 37 °C under anaerobic conditions (MACS1000 Anaerobic Cabinet, Don Whitley, U.K.). Then a single clone was randomly selected and was passed more than 10 times on MRS plates. Morphology observation and Gram staining verified clonal purity. After that, purified bacteria was used for biochemical and molecule identification. The biochemical test results are listed in Additional file 1 Table S1. They showed that these bacteria belong to *Lactobacillus*. For molecular identification, 16 S rDNA primer 27 F (5′-AGA GTT TGA TCC TGG CTC AG-3′) and 1492R (5′-GGT TAC CTT GTT ACG ACT T-3′) were used in this study. BLAST results showed that this *Lactobacillus* was more closely related to *Lactobacillus plantarum* (99% max identity and 96% query coverage)*,* and was therefore named *Lactobacillus plantarum* ZJ316. The 16 S rDNA sequence has been submitted to NCBI with accession number [GenBank: JN126052].

### Antibacterial activity of *L. Plantarum* ZJ316 culture supernatants

The inhibitory effects of the raw extraction of *L. plantarum* ZJ316 culture supernatants were evaluated on 17 bacteria strains (Table 1) using agar plates. An Oxford cup test was selected for this study [54]. In brief, 10 ml of semi-solid medium containing 300 μl of indicator bacteria, such as *E. coli,* was poured onto each agar plate. Then Oxford cups were put on the agar plate. After that, fermented liquid or other activated liquid, such as antibiotic solution, was added to each Oxford cup. After overnight culture, the diameter of the inhibition zone was measured and used as an indicator. In order to reduce possible effects of low pH of the culture supernatants on *E. coli* and *Salmonella*, we adjusted the pH to 6.0 using 1 mol/L NaOH. In addition, the possible inhibitory effects of equivalent acetic acid (200 mM/L) and lactic acid (15 mM/L) were assessed using the solutions of these two acids with pH 3.5.

### Data and sample collection

At the end of the study (day 95), average daily feed intakes (ADFI), average daily weight gain (ADG), food conversion ratio (FCR), mortality and diarrheal rate were calculated for evaluating the effects of different treatments on pig growth. The quantity of food provided and food left over was recorded every day, and the ADFI was calculated as follows: (given feed weight – residual feed weight)/(number of pigs × number of days). The ADG was calculated as follows: (average pig weight at the end – average pig weight at the beginning)/(number of days). The FCR was calculated as follows: (ADG/ADFI). The numbers of dead and diarrheal pigs were recorded every day and the ratios were calculated as follows: (number of dead pigs)/(total number of pigs) and (number of diarrheal pigs)/(total number of pigs). Nine pigs were selected for slaughter at the end of the experiment; and then muscle samples were collected for pork quality evaluation, ileal mucosa samples were collected for morphology observation, and ileal mucosa and cecal contents were collected for bacterial community analysis.

### Evaluation of pork quality

After 60 d of exposure to antibiotics and *Lactobacillus*, three pigs from Group 1, six pigs from Group 2 (three selected immediately after the cessation of the addition of *Lactobacillus*, and three selected one week after halting *Lactobacillus*) were randomly selected for evaluation of pork quality. After slaughter, muscle samples from the *Longissimus thoracis* (LT, located between the 12th and 13th ribs) and fillet were collected for evaluation of the pork quality. A reflectance spectrophotometer (Minolta CM-2002; Osaka, Japan) was used to measure the color at the surface of a 2-cm-thick steak of *Longissimus thoracis* muscle after exposure to air for two hours. The parameters registered were L* (lightness), a* (redness), and b* (yellowness). Each value was the mean of 10 determinations per sample on the same slice, avoiding areas with excess fat.

Muscle pH was measured at 45 min and at 24 hours (starting points from the minute the muscles were removed from the corpse) using a portable pH meter equipped with a glass electrode (Hanna HI 8424, Hanna Instruments, Eibar, Spain). To measure the drip loss, samples were placed on a supporting mesh in a sealed plastic container with no contact between sample and container. Three pigs were selected for sample collection and three muscle samples were collected from each pig. After a storage period of 24 hours and 48 hours at 4 °C, the samples were taken out of the container, dabbed lightly onto filter paper, and weighed again. Drip loss was expressed as a percentage of the initial weight based on Honikel [55]. Marbling scores were calculated according to the NPCC 1999.

Texture profile analysis (TPA) was measured using a TA-XT2 Texture Analyser (Stable Micro Systems, Godalming, U.K.) equipped with a 25 kg load cell. The Texture Expert computer program (version 1.20, Stable Micro Systems) was used for data collection and calculations. Before TPA analysis, samples were vacuum-packaged (DZD-400/2 S, Jiangsu Tengtong Packing Machinery Co., Ltd), placed in a cooler (4 °C, wind velocity of 0.5 m/s) for 7 days, and then frozen on stainless steel trays at -20 °C. At the beginning of TPA analysis, the samples were fast-thawed in tap water (4 h). Then the vacuum was broken and the samples were wrapped in aluminum foil and cooked at 200 °C in a double-plate grill (Sammic GRS-5) until the internal temperature reached 72 °C. After cooking, steaks were placed in a vacuum bag and immediately immersed in an ice bath to stop further cooking. In this study, hardness, stickiness, springiness, chewiness, gumminess, cohesiveness, and restoring force were determined as described by Bourne [56].

### Morphologic observation of gut ileal mucosa

After animals were killed, ileal tissues (approximately 10 cm anterior to the ileo-cecal junction) were harvested and cut into 1.5 cm × 1.5 cm × 0.3 cm pieces for sectioning. The pieces were fixed in 10% formalin after washing with PBS. Then, the formalin-fixed samples were dehydrated in ethanol, cleared with xylene, and embedded in paraffin wax. Sections (6 μm thick) were stained with hematoxylin and eosin and observed using a light microscope (Leica, Germany).

For measurement of villus height and crypt depth, 10 villi and crypts were selected per section using Leica MZ16A software. The villus height was measured from the villus tip to the bottom, not including the intestinal crypt. The crypt depth was measured from the crypt tip to the bottom. An average of 10 villi and crypts per section was expressed as a mean villus height and crypt depth for each pig.

### Analysis of the gut bacterial community

Ileal mucosa (approximately 10 cm anterior to the ileo-cecal junction) and cecal contents (5-10 g) were collected after slaughter. Fresh fecal samples were collected at the beginning and end of the experiment, corresponding to 35, 95 and 102 days of age. Some gut samples collected from Group 1, Group 2-1 and Group 2-2 were used for bacterial genomic DNA extraction. In this study, a bead-beating method was used as previously described [57]. The concentration of extracted DNA was determined using a NanoDrop ND-2000 (NanoDrop Technologies, U.S.), and its integrity and size were checked by agar gel electrophoresis (1.0%). High-quality DNA was then used for polymerase chain reaction (PCR) and denaturing gradient gel electrophoresis (DGGE) analysis. Primer 341 F (5′-ATT ACC GCG GCT GCT GG-3′) and 534R with GC clips (5′-CGC CCG CCG CGC GCG GCG GGC GGG GCG GGG GCA CGG GGG GCC TAC GGG AGG CAG CAG-3′) against the V3 region of the 16 S rRNA genes were used in this study. The PCR program included 20 touchdown cycles (65 °C-55 °C); followed by 5 cycles of 94 °C for 1 min, 55 °C for 1 min, and 72 °C for 1 min followed by extension at 72 °C for 10 min. Reconditioning PCR was performed before DGGE. DGGE was then performed on a Dcode^TM^ universal detection system (BIO-RAD Laboratories Inc, U.S.) with 8% polyacrylamide gels (ratio of acrylamide to bisacrylamide, 37.5:1) at 60 °C. The gels were electrophoresed at 200 V for 4 h, and then stained with SYBR GREEN І. The bands were visualized and analyzed with Quantity One software (Version 4.6.1; BIO-RAD Laboratories Inc, U.S.) using a match tolerance of 2%.

### DGGE band sequencing

DGGE band sequencing was carried out according to Li et al. [58]. The stable bands in the DGGE gels verified as single bands were excised and eluted in 30 μl TE buffer (10 mM Tris and 1 mM EDTA, pH 8.0). The supernatant after centrifugation (12,000 rpm, 5 min, 4 °C) was used for 16 S rDNA-V3 amplification with the V3 primers 341 F and 534R without GC-clamp using the same program as Li et al. [58]. The amplification 16 S rDNA-V3 segments were cloned into a PMD18-T vector after being purified with a Biospin Gel Extraction Kit (Bioer Technology co., Ltd., Japan). The positive recombinants were screened on 5-bromo-4-chloro-3-indolyl-b-D- galactopyranoside (X-Gal), isopro-pyl-b-D-thiogalactopyranoside (IPTG) and ampicillin indicator plates by color-based recombinant selection. Positive clones were selected for sequencing using an ABI 3730 DNA Sequencer (U.S.) with M13 primer at the Beijing Genomics Institute (BGI, China). In all, 34 DGGE bands were sequenced and most closed sequences were identified using a BLAST search.

### Short-chain fatty acids (SCFAs) analysis

Fecal samples (0.5 g) were dissolved into 10 ml phosphate buffered saline (PBS), vortexed, and then centrifuged at 12,000 × g for 10 minutes at 4 °C. Then the supernatants were filtered through a 0.45 μm membrane filter for HPLC detection. In this study, the levels of acetic, butyric, citric, formic, isovaleric, lactic, malic, propionic, and tartaric acids were investigated. A Prevail^TM^ Organic Acid column (250 mm × 4.6 mm) was used with the detection conditions: temperature, 40 °C; wavelength, 217 nm; pressure, 0.1-4,000 psi.

### The inhibitory effects of mequindox on *L. Plantarum* ZJ316

An Oxford cup test was executed as described above, and the inhibitory effects of 50 μg/ml, 100 μg/ml, 250 μg/ml, 500 μg/ml and 750 μg/ml of mequindox were evaluated.

### Statistical analysis

To analyze growth, each pen from each group was considered a single replicate. For meat color, pH value, drop loss and marbling score, each pig from each group was considered a single replicate. For meat TPA analysis, three samples were collected from each pig and used as replicates. For gut villus height and crypt depth analysis, 30 values were collected from each group and used as replicates. All of the above data were statistically analyzed using the One-Way ANOVA program included in the statistical software package SPSS 13.0 (IBM, U.S.). Least-significant difference (LSD) was selected for post hoc multiple comparisons. For fatty acid analysis, fecal samples were collected from pigs that had been selected for meat quality evaluation and were considered as replicate. They were then analyzed using the means program included in the statistical software package SPSS 13.0 (IBM, U.S.). All values were presented as mean ± standard error. p < 0.05 was considered significant.

## Authors’ contributions

CS performed the pig growth experiment and carried out the data analyses; YY designed the experiments, carried out data analyses, and drafted the manuscript; XW performed the pork quality experiment; XL performed the inhibitory experiment of antibiotic; DS isolated the Lactobacillus strain and assisted with the pork quality experiment; XW designed the experiments and revised the manuscript; QG designed the experiments, carried out data analyses, and drafted the manuscript. All authors read and approved the final manuscript.

## Supplementary Material

Supplementary Table 1 Fermentation pattern of *L. plantarum *ZJ316*. Supplementary Figure 1. Inhibitory effects of the *Lactobacillus plantarum* ZJ316 culture supernatants at pH 6.0. Culture supernatants of *L. plantarum* ZJ316 were collected and adjusted to pH 6.0 using 1 mol/L NaOH. The inhibitory effects were evaluated on *Escherichia coli* and *Salmonella* using agar plates. A: Inhibitory effects of the culture supernatants on *Escherichia coli*. B: Inhibitory effects of the culture supernatants on *Salmonella*. Supplementary Figure 2. Comparison of the inhibitory effects of culture supernatants of *Lactobacillus plantarum* ZJ316 with acetic acid and lactic acid at pH 3.5. Salmonella was used as indicator bacteria for comparing the inhibitory effects at pH 3.5. 1, represents culture supernatants of *Lactobacillus plantarum* ZJ316 was added into the Oxford; 2, represents lactic acid was added into the Oxford and 3, represents acetic acid was added into the Oxford. Click here for file
