# Supplementary material for: Effects of lactobacillus plantarum ZJ316 on pig growth and pork quality
Source: BMC Vet Res. 2012 Jun 25;8:89. doi: 10.1186/1746-6148-8-89 (PMC3482153; doi:10.1186/1746-6148-8-89)

Supplementary Table 1. Fermentation pattern of *L. plantarum* ZJ316*

Substrates	Activate 24 h	Activate 48 h
Amylum	-	-
Arabinose	+	+
Cellobiose	+	+
Esculin	-	-
Glucose	+	+
Glutin	-	-
Hydrogen sulfide	-	-
Indole	-	-
Lactose	+	+
Maltose	+	+
Mannitose	+	+
Mushroom polysaccharide	+	+
Olein	-	-
Peptone Water	-	-
Raffinose	-	-
Sucrose	+	+
Urea	-	-
Xylose	+	+

*, + represents positive reaction; - represents negative reaction.

Supplementary Figure 1. Inhibitory effects of the *Lactobacillus plantarum* ZJ316 culture supernatants at pH 6.0. Culture supernatants of *L. plantarum* ZJ316 were collected and adjusted to pH 6.0 using 1 mol/L NaOH. The inhibitory effects were evaluated on *Escherichia coli* and *Salmonella* using agar plates. A: Inhibitory effects of the culture supernatants on *Escherichia coli*. B: Inhibitory effects of the culture supernatants on *Salmonella*.

A. Inhibitory effects on *Escherichia coli*

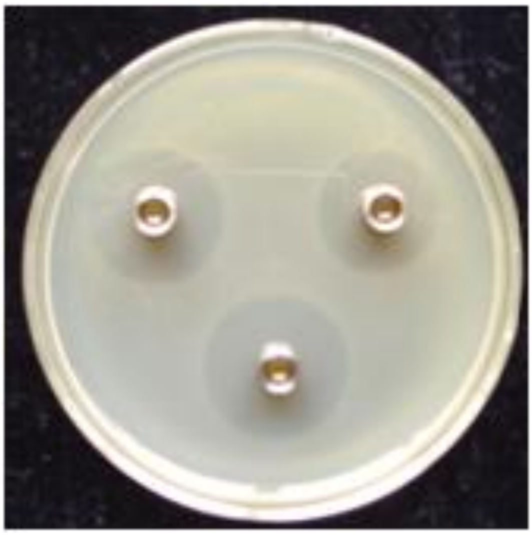

B. Inhibitory effects on *Salmonella*

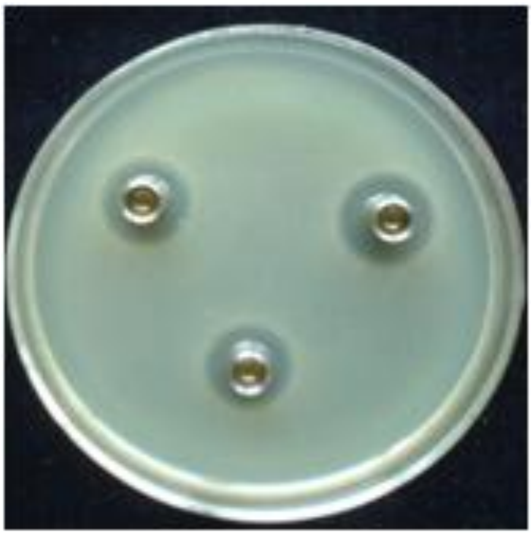

Supplementary Figure 2. Comparison of the inhibitory effects of culture supernatants of *Lactobacillus plantarum* ZJ316 with acetic acid and lactic acid at pH 3.5. Salmonella was used as indicator bacteria for comparing the inhibitory effects at pH 3.5. 1, represents culture supernatants of *Lactobacillus plantarum* ZJ316 was added into the Oxford; 2, represents lactic acid was added into the Oxford and 3, represents acetic acid was added into the Oxford.

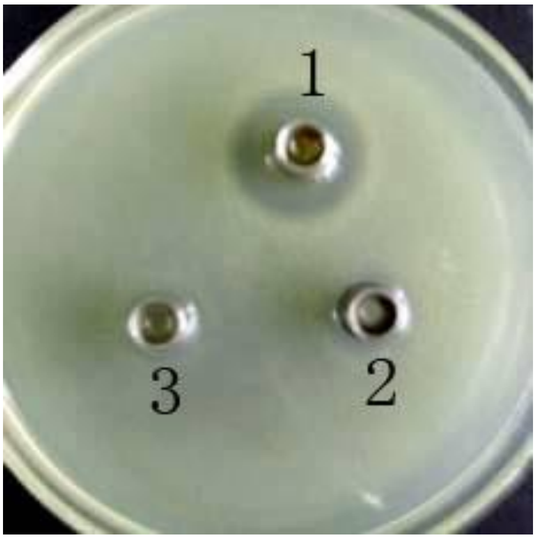

Supplement: Supplementary Table 1 — Fermentation pattern of L. plantarum ZJ316*. Supplementary Figure 1. Inhibitory effects of the Lactobacillus plantarum ZJ316 culture supernatants at pH 6.0. Culture supernatants of L. plantarum ZJ316 were collected and adjusted to pH 6.0 using 1 mol/L NaOH. The inhibitory effects were evaluated on Escherichia coli and Salmonella using agar plates. A: Inhibitory effects of the culture supernatants on Escherichia coli. B: Inhibitory effects of the culture supernatants on Salmonella. Supplementary Figure 2. Comparison of the inhibitory effects of culture supernatants of Lactobacillus plantarum ZJ316 with acetic acid and lactic acid at pH 3.5. Salmonella was used as indicator bacteria for comparing the inhibitory effects at pH 3.5. 1, represents culture supernatants of Lactobacillus plantarum ZJ316 was added into the Oxford; 2, represents lactic acid was added into the Oxford and 3, represents acetic acid was added into the Oxford. [file 1746-6148-8-89-S1.pdf]
